# How neurosurgeons maintain and update their professional knowledge in a self-directed learning context

**DOI:** 10.1186/s12909-024-05692-9

**Published:** 2024-07-16

**Authors:** Jodie Freeman, Andreas Raabe, Felix Schmitz, Sissel Guttormsen

**Affiliations:** 1 Department of Neurosurgery, Inselspital, University Hospital Bern, Bern, Switzerland; 2 Institute of Medical Education, Medical Faculty, University of Bern, Bern, Switzerland

**Keywords:** Self-directed learning, Health professionals, Learning strategies, Digital tools

## Abstract

**Background:**

Given the changes in the current learning environment health professionals are facing major challenges to keep up with current and updated information with the rapidly growing clinical and scientific knowledge base. Being able to identify relevant, high-quality articles, adapt or adopt to new learning strategies with an already intense workload are just a few of the main challenges. Self-directed learning is a key skill of competent health professionals and describes the process by which individuals evaluate their learning needs, goals and the resources needed for learning, however the emerging problems for professionals practicing SDL are manifold.

**Design:**

A qualitative, exploratory approach based on four research questions was used to understand how skilled neurosurgeons maintain and update their professional knowledge. Twenty-six neurosurgeons within the University Hospital of Bern completed a semi-structured interview.

**Results:**

One of the main findings concerns the differences between neurosurgeons regarding the SDL strategies they employ, which is compounded by their level of experience. All participants recognized that new or alternative learning approaches are necessary to manage the learning landscape, and for many this concerned their use of learning digital tools. Many, however, were unsure how to change their current behavior.

**Conclusion:**

The results highlight that positive factors influencing SDL in the workplace include learning leadership and support in identifying new or alternative strategies, an internal culture committed to learning as well as digital learning tools and networks. All are vital in managing the continuously evolving learning environment.

## Introduction

The expectation to maintain and update knowledge as a health professional is critical in the medical field in order to provide the best patient care [1]. Medical professionals face major challenges to keep up to date with the rapid expansion of the clinical and scientific literature [2]. Medical knowledge has grown exponentially over the last 10 years and this is anticipated to continue [1]. In the biomedical field alone, more than 1 million papers are added to the PubMed database each year which equates to two papers a minute [3]. This situation affects professionals in the healthcare system, such as practitioners, patients and systematic reviewers, but is also a barrier to the adoption of evidence-based medicine [4]. High-quality online evidence-based information is often mixed with lower quality information [4], thus making it difficult for health professionals, especially those with less experience, to identify relevant high-quality evidence-based resources. Despite the exponential growth in academic papers [5], health professionals are required to stay up to date in their respective fields and maintain their existing knowledge base. To provide the most up to date patient care and manage the information load, health professionals must commit to lifelong learning after their formal education [6].

Recent research has identified Self Directed Learning (SDL) as the most appropriate methodology for an individual to achieve and maintain their levels of expertise [7]. Knowles, 1975, p.18 describes SDL as “*a process in which individuals take the initiative, with or without the help of others, in diagnosing their learning needs, formulating goals, identifying human and material resources for learning, choosing and implementing appropriate learning strategies, and evaluating learning outcomes*” [8]. The increased importance of SDL and skill development is prominent in medical education [9] and many courses offer support in information literacy, learning tools and SDL strategies [10, 11]. However, despite such SDL training, the challenges for professionals practicing SDL are manifold. For example, the traditional means of managing information (e.g., individually storing data in electronic filing systems) are ill suited to the realities of the digital age [12]. Information skills training has become a prominent aspect of evidence-based training in medical education, however the impact of this training requires further evaluation [13], particularly in relation to SDL. Workload and the changing nature of healthcare provision make it increasingly necessary for health professionals to use their time productively and to learn efficiently [14].

The explosion of health care knowledge worldwide and the increasing access to both relevant information as well as misinformation is higher than that for any previous generation in history, particularly via social media platforms [15]. Therefore, it is necessary for individuals conducting SDL to be able to select relevant, high-quality articles from many sources. This suggests that health professionals need to adopt new strategies or approaches to meet the demands of the current ever-growing learning environment. Habit theory highlights that habits are routine, automatic behaviors that are repeated regularly [16], and are considered a learned experience. A habit forms when behaviors are repeated in a stable context [17]. This suggests professionals, especially experienced professionals, may find it difficult to break habits previously successfully employed to select information [18].

The literature indicates the challenges faced by health professionals regarding learning have led to an increased workload. Firstly, many health professionals need to invest a significant amount of time to find a more efficient approach to conduct SDL. Secondly, as it can be difficult to identify the most relevant articles, many professionals attempt to read as many articles as possible in an attempt to avoid missing important papers [14]. This is common with less experienced health professionals and often leads to a gap between intention and behavior [19]. Therefore, individuals may not only need to break habits but also to retrain in new strategies/ technologies to manage information overload and support personal learning. More support for learning could be provided in the work environment, such as a communal website, to share information, intra-departmental tools, feedback from experts [20] or granting time for personal learning.

According to intention-behavior gap theory, it is impossible to read all relevant articles despite having good intentions [19]. Studies have shown that this increase in workload or cognitive exhaustion has led to a decrease in performance, the ability to process, and for some, in coping with the volume of information [21]. Moreover, the realization of the impossibility of keeping up with all the relevant material can completely demotivate some learners [22] and can lead to a sense of ‘learned helplessness’. Learned helplessness is a motivational problem that can arise when someone feels as though they cannot control their environment and subsequently impairs their ability to learn [23].

An important aspect of SDL is to employ strategies that motivate and facilitate learning [24]. It is key for health professionals to identify their own learning needs and to understand the relationship between individual workloads, motivation, habits and learning strategies in the context of SDL [4]. In addition to traditional learning strategies such as a good organizational system, prioritizing tasks and seeking feedback from peers [6, 25], more recent research highlights the use of electronic mediums such as digital libraries and recommended reading lists based on previous searches [26]. Health professionals also report the use of alternative methods to conduct their SDL such as journal clubs and interdepartmental communication tools [27]. Studies have confirmed journal clubs support, and are considered effective by learners, in their continued education [28] by facilitating knowledge gain and retention, skills of manuscript writing and critical appraisal. Recent research has found that professionals are utilizing specialized learning tools designed specifically to support learning and document management [29], such as Mendeley, Evernote [30] and more recently, Notion [31]. These tools offer a variety of functions to help motivate, increase productivity, reduce workload and support learning [32], such as information storage, task management and project tracking [30, 31].

Although it appears the digitalization of learning is increasingly common place, it can be a struggle to identify which digital research tools are most the suitable, including what functions they offer or how to use them appropriately [33]. From this perspective, medical experts must have an awareness of such technology to benefit from the range of tools available [34]. Research suggests that many professionals avoid new technologies following unsuccessful experimentation which unfortunately led to confusion, wasting of time and learned helplessness [35]. Despite the increased current use of tools, the vast majority of health professionals feel insufficiently trained to deal with the digital revolution [36]. As a result, many tend to remain with their trusted traditional methods [35] and believe themselves unable to navigate the complexities of a new digital tool.

Within the literature there have been many studies exploring SDL by health professionals [37]. SDL has been particularly well explored in medical education [38], however, studies focussing on SDL by neurosurgeons has been limited. Previous studies have highlighted the impact of the internet and technological tools on the continued learning of this group [39]. This research acknowledges the importance of identifying useful, relevant and above all reliable information. There are, however, no current studies exploring the strategies and use of digital tools which neurosurgeons use to continue in their SDL. This study aimed to investigate how neurosurgeons overcome the challenges of the current learning landscape to maintain and update their professional knowledge outside of their organized training, in-house curriculum or formal continual education. Specifically, we focus on the process of neurosurgeon SDL in relation to their self-directed personal learning strategies and use of digital tools to overcome the challenges of the current environment.

### Research questions

When using the term SDL, this paper will refer to the definition by Knowles (1975) and SDL which is conducted outside of organized education. Derived from the background described above we formulated the following research questions for this qualitative study.

*RQ 1: how do neurosurgeons maintain and update their professional knowledge in an SDL context?*

*RQ2: How do the demands of the current learning environment, combined with an intense workload, impact the motivation of neurosurgeons in their SDL?*

*RQ 3: What strategies and/ or digital tools are neurosurgeons using to navigate the current learning environment and support their SDL?*

*RQ 4: What are the challenges neurosurgeons face in relation to the use digital health tools to support their SDL?*

## Methods

The research questions were addressed from a qualitative, exploratory approach, utilizing semi-structured interviews to gain an understanding of how skilled neurosurgeons maintain and update their professional knowledge. This qualitative study was guided by the consolidated criteria for reporting qualitative research (COREQ) [40].

### Study setting

The study was conducted within the University Hospital of Bern and spanned a wide breadth of experience in practice. The interviews took place in the Neurosurgery Department, in a quiet office to ensure confidentiality and minimize distraction. The interviews were conducted during the workhours of the surgeons and were pre-arranged a week in advance to ensure sufficient time was allocated to conduct the interview and that the surgeon was less likely to be on call. All necessary steps were taken to complete the interview in one session.

### Study sample

We investigated neurosurgeons in this research, which were identified as a population of health professionals with an existing hectic schedule and who are required to maintain their knowledge and keep on top of the latest research.

Twenty-six neurosurgeons participated in the study. Theoretical saturation [41] was reached while interviewing the last six surgeons, therefore it was decided to stop recruitment at twenty-six.

Ten participants were female (M = 35.5, SD = 6.11), sixteen were male (M = 34.9, SD = 8.13), aged between 27 and 54 years old. Twelve were less experienced surgeons (46.2%) and fourteen experienced surgeons (53.8%). Surgeons were classified as less experienced if they had nine years or less as a certified doctor [42], whereas more experienced surgeons had at least ten years or more experience at the time of the interview.

Neurosurgeons were recruited as an opportunistic sample [43] as the project was situated in this speciality and access to this population was readily accessible. Neurosurgeons are distinctive in comparison to other health professionals due to their length of training [44], required skills and expertise [45], high workload [44], high complexity [45], and the rapid evolution and adoption of new developments [44]. These characteristics have an impact on their ability to conduct SDL [37]. Murad et al. found SDL to be effective in improving learning outcomes in health professionals and advanced learners, as defined in the study to include doctors, who undertook SDL better than other health professionals.

### Development of instrument

The interview schedule was developed in seven steps and followed the process described in Gideon (2020). (See Appendix 1 for a detailed description). The interview questions were based on ten sub-themes based on the SDL literature (Table 1). The final interview schedule consisted of a natural flow of thirty-three questions (Appendix 1).

**.**
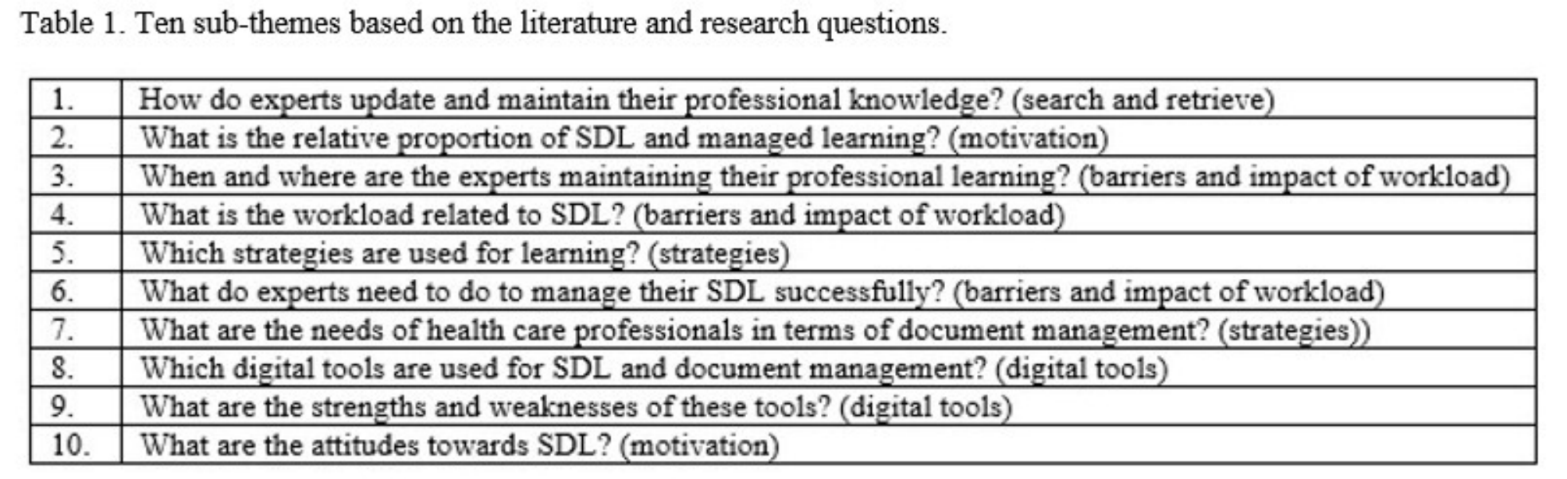
.

These ten sub-themes were used as the basis for the five initial concepts that guided the analysis (see Table 2). The ten sub-themes were based on aspects of SDL literature which differed in complexity, amount of information and the experiences of the research team. The development of these sub-themes was guided by the inductive analysis process by Witkowsky and Bingham (2021) [46]. Each of the ten sub-themes corresponds to one of these five concepts as can be seen in Table 1.

**.**
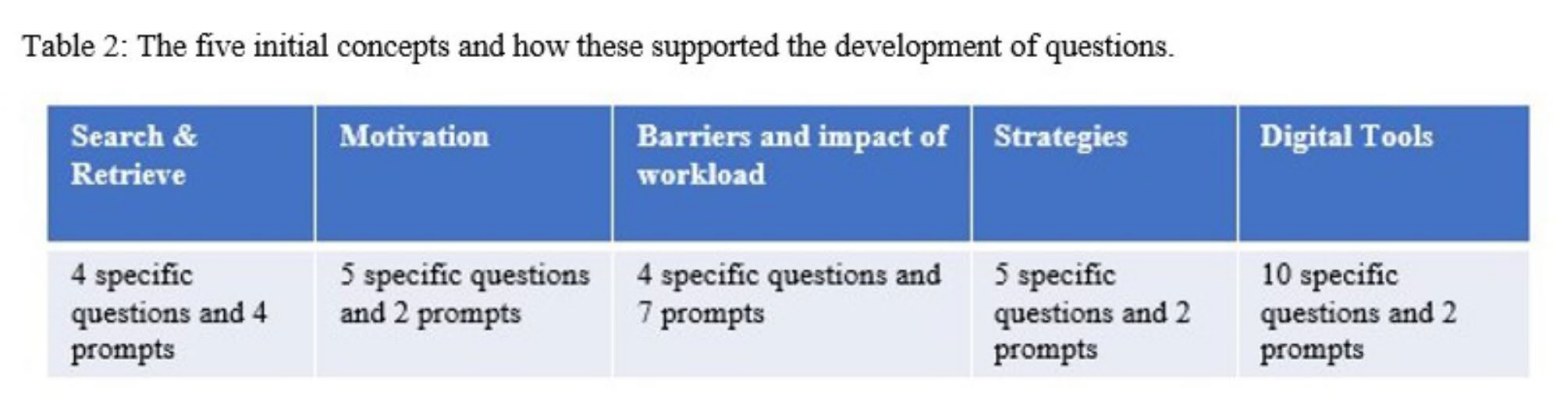
.

The complexity and quantity of literature relating to these five concepts differed and the number of interview questions relating to each theme reflected this. For example, digital tools required ten interview questions to gather the necessary information, in comparison to ‘search and retrieve’ which only required four questions. Overall, twenty-eight of the questions were guided by these initial themes. An additional five questions were posed to build rapport and to help ensure no additional information was missed.

### Data collection

An invitation email was sent out to surgeons in the Neurosurgery Department in Bern (Appendix 2). The interviews lasted between 21 and 86 min. In line with COREQ guidelines [40], all interviews were audio recorded. Data were collected between September 2019 and December 2019. The main researcher conducted the interviews and was independent of the neurosurgeons as guided by the COREQ checklist [40]. In addition, to encourage transparency, the aim and overall purpose of the study was clarified, and the final interview guide was clearly visible (see appendix 1). Notes were made during the interviews and these field notes were included during the coding phase and reviewed independently.

To ensure reflexivity, the main researcher (JF) clarified interviewee identity, credentials, occupation, experience and training before the main questions [47]. The experience and training of the researchers was taken into consideration during the creation of the questionnaire [48]. To limit bias, the team also referred to the literature on SDL to develop and create the final interview instrument. Moreover, the main researcher did not work closely with the neurosurgeons, was independent and this encouraged frank discussions [49]. It was highlighted to participants that when referring to SDL this did not include the organized continuing education they attended, but instead to SDL conducted outside of their organized education courses.

### Analysis

**Fig. 1:**
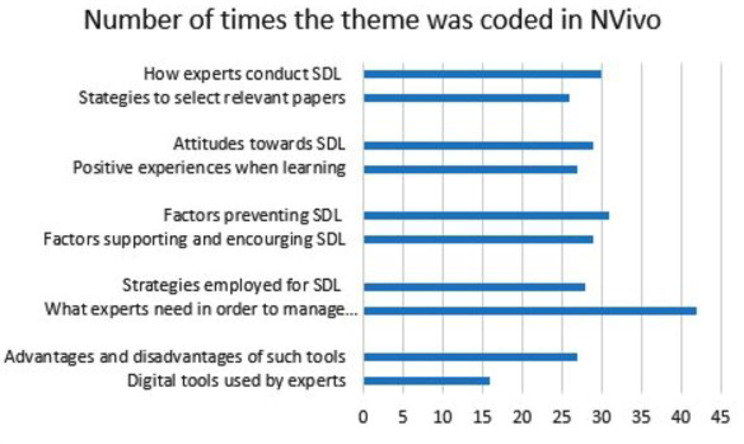
The ten initial main themes coded in NVivo

All the interviews were transcribed according to the intelligent verbatim transcription method (McLellan et al., 2003) [50]. The interviews were initially transcribed using a software program and then manually proofread by the main researcher. Data were coded and reviewed by two independent coders and guided by NVivo. Participants were coded by the order in which they were recruited and by the year they passed their board certificate. Interviews were analyzed using thematic analysis according to the deductive and inductive approaches of qualitative analysis as set out by Witkowsky and Bingham (2021) [46], and guided by NVivo (Bazeley and Jackson, 2019). In the initial coding phase, and to the guide analysis, the research team identified five initial concepts (as shown in Table 2). The researchers read and analyzed the data multiple times allowing codes to emerge. This form of inductive analysis used the participants’ own words (in vivo coding) and constant comparative analysis to identify patterns and emerging themes guided by Witkowsky and Bingham (2021) [46]. Through this process and further in-depth analysis of the data, ten main theme nodes were identified (see Fig. 1). These theme nodes were further refined in subsequent rounds of data analysis. Several rounds of coding were performed, each time focusing on identification of new themes and amalgamation of subsidiary nodes.

The ten themes shown in Fig. 1 refer to the codes that appeared or were discussed most in the data and after several rounds of analysis were so grouped. The themes were grouped according to the emerging SDL topics. On further analysis and with discussion between coders, it became clear that some of these ten themes had similarities with each other in the context of SDL and they were then condensed into the five main themes. Figure 1 shows that the first two codes (one and two) are similar and these therefore were amalgamated into one of the themes, ‘selection of sources’, the third and fourth codes were amalgamated into ‘attitude and motivation’, the fifth and sixth codes were combined into ‘supporting and preventing factors’, the seventh and eighth codes amalgamated into ‘strategies to support SDL’ and finally the ninth and tenth codes were amalgamated into digital tools to support SDL.

These final five themes gave new insights (inductively from the data) and supported some expectations derived from the SDL literature. Each of these five inductive themes were guided by the four overall research questions.

### Ethical considerations

All participants were provided with an information statement. They were assured anonymity and confidentiality to encourage honest, open discussions on their learning approaches and practice. Interviews were conducted in a private office space with only the main researcher. Completion of the interview was considered to represent informed consent. The need for ethical approval is waived by the ethics committee of *Kantonale Ethikkomission Bern* (Swiss ethics committee on research involving humans).

### Findings

**Fig. 2:**
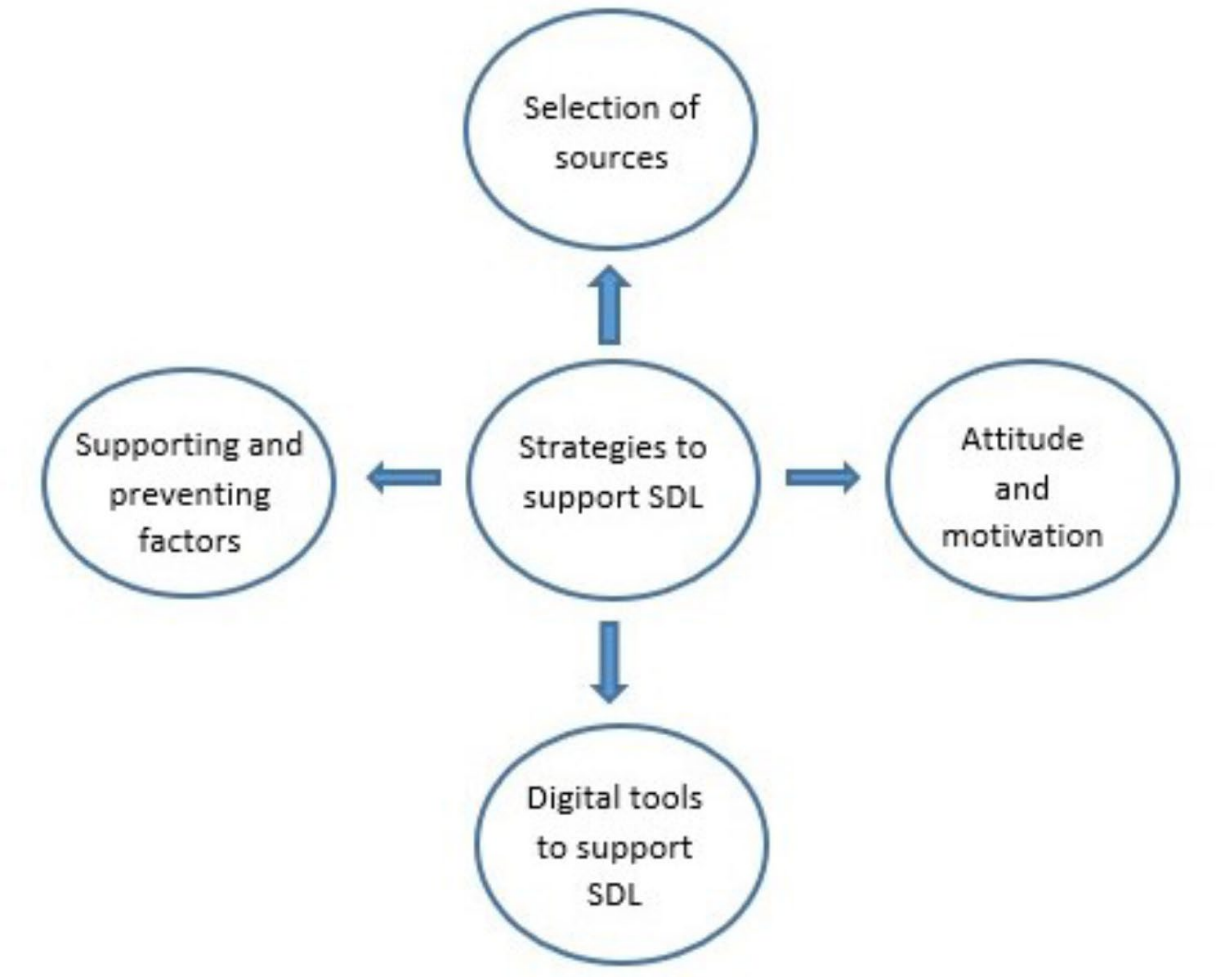
The five themes identified from the data

This study sought to investigate how neurosurgeons maintain and update their professional knowledge in a self-directed learning context, focusing on their learning strategies and use of digital tools. Five themes identified from the analysis were: strategies for SDL and selection of sources, attitude and motivation towards SDL, factors supporting and preventing SDL, strategies to support SDL and digital tools to support SDL (see Fig. 2).

Five themes were identified from the interview data, however throughout the discussions it was evident that the strategies to support SDL used by the surgeons underpinned the other four themes. How they selected sources, overcame barriers and challenges, chose digital tools and maintained motivation were intertwined with the strategies they used to conduct SDL. Therefore, the theme, strategies to support SDL is the theme that underpins each of the other four themes and is reflected in the diagram.

To update and maintain knowledge, all surgeons utilized specific medical or academic internet search engines such as Pub med, OVID, Medbase etc. Despite using key terms and filters, search queries often resulted in an insurmountable number of articles. To identify relevant articles, surgeons needed scroll through many, sometimes hundreds of search results. In many cases, the number of “relevant and irrelevant articles” was unmanageable.

*Sometimes it will take me half an hour just to read through the list of abstracts of all the articles from just one search query. They hardly come back in any order of priority. (0172018)*

Less experienced surgeons reported more issues with selection than experienced surgeons, stating that their main strategies relied on the use of filters and key words. However, as the amount of available research increases and in the absence of a better approach, they were open to new strategies such as information pooling.

*It’s not always easy to know what to read, I often just use the sources that are recommended in the clinics. (*006312014)

*It would be great if we could find and share relevant articles with each other, it would definitely cut the workload down. (018312016)*

Less experienced surgeons said they benefitted from the advice and recommendations of more experienced colleagues but that it would be more useful to share the most up to date relevant findings with each other as they often need to research the same topics. It was suggested that a specialized platform or website with articles only relating and available to neurosurgery would be more efficient.

*It would be great if we had a place, like a platform where only neurosurgeons recommended articles where only relevant findings were available. (019392014)*

More experienced surgeons acknowledged that their knowledge and experience make this easier, as it’s a habit formed over time to select quickly required articles. They conclude that the ability to draw on a previous knowledge base to select relevant articles reduced the amount of time to complete tasks.

*I often know what I’m looking for and I have my own personal markers – otherwise, how do you differentiate between an author who’s not very relevant but is screaming very loud, with many citations and one who’s not. There are so many factors to consider I guess you just learn to spot them with experience. (014402020)*

*I can identify quickly if it’s worth reading, I have some kind of flow chart, a check list in my mind. (002402004)*

However, it was also noted that some relied heavily on “old habits” and that this was not always effective for identifying the most up to date relevant research.

*I think I mostly find what I’m looking for, either in my conference folder or my congress folders. I’m not sure. (026492004)*

### Attitude and motivation

Findings show that how a surgeon selects their documents alongside the learning strategies they employ has an impact on their motivation to conduct SDL. In contrast to ten years ago when time could be allocated every month to read all relevant papers, many experienced surgeons now accept this is impossible and they instead stick their personal strategy.

*I don’t even open the emails anymore, I just read the titles of the journals of the month. It’s far too many to be expected to read. They should summarize all the most important journals that month into a paragraph. (026492004)*

*I have 20 notifications with apparently new and relevant findings, I don’t even have time to read the notification. (016542000)*

However, the neurosurgeons do seem to be more confident the articles they do have time to read are the most important or relevant. This relates to their selection strategy; they rely on certain markers based on habit and experience.

*I just seem to know what I’m looking for, its second nature. (026492004)*

In contrast, some less experienced surgeons who are overwhelmed by the sheer number of articles admit their strategy was often to read as many possible, and they were initially motivated to do so in case they missed something. Indeed, they often used many hours of free time to catch up on necessary research.

*Well, I’m still new, so I’m not really maintaining, just updating. I try to read everything I can, so it means I read a lot after work. I usually spend my days off catching up on research journals (012262018)*.

Some less experienced surgeons also indicated that despite planning to learn at certain times of the day or week, the amount to read was frequently so overwhelming it was impossible to know where to start. Instead of scaling down the task to make it more manageable, they became completely demotivated and didn’t make any attempt to study the articles. Accompanying this absence of motivation was often a sense of guilt and failure.

*I had all these plans last weekend to catch up on all the reading I’ve missed. I had so many articles in my unread folder I didn’t even know what topics I wanted to start with. In the end I didn’t read any. (013312016)*

*I haven’t had a chance at all this week to look at any of the stuff I saved and now I’m already on another topic so I’m not sure I’ll even have the chance again. (006312014)*

### Factors supporting and preventing SDL

The workload for all surgeons is undoubtedly high, however, our findings highlight that the impact of an inability to select papers or topics in the face of an increasing number of articles affected less experienced surgeons much more than their experienced peers. All less experienced surgeons reported that their SDL is done outside of their working hours with most indicating as much as 95% SDL was conducted outside of work.

*If I’m lucky I can get some time between rounds, but you always have to be available so it’s not the right environment to sit down and read long articles. (018312016)*

In addition, if a lack of motivation had reduced learning efforts, then the pressure and workload to catch up was increased for the next planned SDL session.

*I didn’t do anything last weekend; I was just too tired. I will have to make up for it on my next day off. (017272018)*

In contrast, more experienced surgeons shared most of their updating and maintaining knowledge took place during working hours except for more significant research demands.

*I just needed to find some evidence to support the review I’m writing, I can usually find some time. (021402006)*

Moreover, this group found they could create time in the working day by delegating everyday tasks, but they were not necessarily happy to do this.

*It’s frustrating because you need to keep updated in your career but even if you’re in a position to delegate the work, there’s always this dissatisfaction because it’s not always a good solution (014402010)*.

### Strategies to support SDL

Within the theme of strategies to support SDL and source selection, study participants shared how and where they stored their articles. Findings revealed that twenty-one out of twenty-six surgeons used their computer hard drive as their main document repository as it was simple to use, and they were familiar and used to it. However, many reported that as the amount of information increased, it became difficult to retrieve information in a timely fashion. Despite being organized, users could not always find articles using key words. Many reported that this system resulted in many disorganized folders and subfolders containing multiple files.

*I guess it was after my exam that I realized I couldn’t just keep storing papers into different folders. I had so many in my unread box that I could never find anything again. (012262018)*

Many experienced surgeons stated that they have used the same search, storage, and retrieval strategies for a long time, however some admitted this could often be a time-consuming process when trying to locate dated articles.

*I can usually find what I need in my congress or conference folders but sometimes there are so many papers, or I can’t remember what I saved it as. (016542010)*

Of the five surgeons who reported employing a Document Management (DM) system outside of their hard drive, three used the more advanced document management systems such Evernote or Mendeley. Two less experienced surgeons stated they relied on Notion, a digital tool that provides a range of functions alongside DM.

The findings highlighted that there were differences between the attitudes of more and less experienced surgeons towards the possibility of a using an alternative DM system. All reported concerns about the time and effort needed to use a new strategy/tool without knowing if it’s suitable.

*I use my computer; I mean I basically only used my hard drive but I’m struggling to find things and we have so many new articles to save. I downloaded Mendeley, as someone recommended it, but it’s a bit of a project, I’m not sure how it works yet. I hope it’s worth it. (003282016)*

However more experienced surgeons had more concerns over usability, complexity and how much technical prowess is needed to navigate such tools. Despite admitting these learning tools may be useful, they preferred to stay with their existing strategies.

*I think it would take me longer to learn how to use it than it does to use my computer. I’m not saying I wouldn’t try it, but I don’t know much about what’s available. (023372007)*

Two less experienced surgeons reported using a new research tool which also stores documents, called Notion. Both stated that they had evolved with the advancement of technology and tend to try the newest tools.

*I use Notion. It gives you the possibility to define a topic and then save all types of media and documents and everything in between in the same place. It’s just like freehand and it’s awesome because you can save everything on that topic. (020332014)*

### Digital tools to support SDL

This section focuses on the digital tools’ neurosurgeons use to support their SDL. These tools range from the “usual suspects” such as Microsoft Windows, Dropbox, Google, etc. Some of the less experienced surgeons reported using digital tools specifically designed to support research activities such as Notion.

The findings highlight that more experienced, and some less experienced surgeons, prefer to use more traditional methods of learning, stating that these strategies represent electronic versions of the traditional research ways.

*I much prefer to go through articles and highlight them with a pen, I find that I remember the information more anyway. I have a colour system which I’ve used since university so it’s the same thing really. (022272017)*

Many surgeons stated that they hadn’t heard of most of the digital tools available to support learning and therefore had not thought about using them.

*I’m not even sure what tools there are to help me research, and I don’t have time to find out. (attribution ref)*

In some cases, despite not being entirely happy with their learning strategies, more experienced surgeons seem less motivated to try digital research tools as they would not be intuitive to use, preferring to “keep things simple.” They seemed afraid it would be too complicated and wanted to avoid wasting time.

*I tried Mendeley recently, but I never got into it, I’ve heard of some others, but I don’t have the time to learn how to use them and who even knows if they work. I guess if it was a guarantee that it was already a good system. (*017272018)

In contrast, some less experienced surgeons highlighted that they were open to try the latest technologies to support their research activities however they often weren’t aware of the tools nor their functionalities. Nevertheless, they had no time to explore available digital tools.

*I hadn’t even heard of Notion until today, I maybe would try it, what it does. I should stop printing out everything, so I need to do something, I just don’t have the time. (019392014)*

Less experienced surgeons reported using specialized tools to help them highlight, tag key words and efficiently structure and compile a research article.

*I’ve always had problems starting to write on a blank page, with the program I’m using now it sets out the structure and includes all the information you’ve highlighted into each section even before you’ve started. (011322013)*

*I love Notion, it helps me organize my notes and all my diagrams and well everything. (005332010)*

Our findings show that surgeons who are ‘digital natives’ i.e. those who have grown up with modern information technologies often utilize a combination of the tools to support them in their learning.

*I use Notion, I don’t know if you have heard of it and used to work with Evernote, and I use Endnote and a couple of medical apps. I changed to Notion, and I’ve been quite happy with it.* (*022272017)*

These surgeons also shared their familiarity with information technologies was a learned skill informed by their educators and peers.

*I used Evernote at Uni, my supervisor recommended it. Then I just tried other ones that my friends were using. The latest one I tried was Notion. (022272017)*

## Discussion

Guided by the SDL literature and the research questions, five inductive themes relating to the SDL of neurosurgeons emerged from the data. The differences observed between more and less experienced surgeons within and across each of the themes was particularly informative. The first research question seeks to understand how neurosurgeons perform SDL. There were differences in how SDL is conducted, in that surgeons with more experience had their own effective strategies to quickly identify relevant information. These strategies were based on their previous experience such as, knowledge of the author and the depth of the findings. However, such habituated behaviour [51] may lead to an inability to adapt to the increasing amount of information and ultimately may be detrimental to their SDL.

In contrast, our findings revealed less experienced surgeons who cannot rely on ingrained habits reported how an inability to efficiently select relevant articles affected their ability to conduct SDL. This group were much more open to learning new strategies. Some less experienced surgeons highlighted the benefits of information pooling and advocated more of a community-based approach to share workload and learnings among colleagues. Recent academic medical blogs have similarly suggested learners should team up and share relevant knowledge to stay on top of studies to avoid missing important papers [20]. Such a platform of global mentor-peer support could help ensure less experienced surgeons would not miss landmark research.

Our second research question focused on how the learning environment combined with an intense workload affects the motivation to conduct SDL. Findings showed there were differences in both attitude and behavior between more and less experienced surgeons in relation to their motivation to learn. More experienced surgeons accepted that it is impossible to read all articles and therefore did not even attempt to. This group instead relied on their strategies to select relevant articles choosing to “read smart” not read everything. However, as medical knowledge is increasing exponentially [1] these current strategies may become more and more ineffective.

In contrast, less experienced surgeons felt the need to read as many articles as possible in case they missed something important. Within the literature, the difficulty of fulfilling such a task is often reported to lead to feelings of failure and ultimately a lack of motivation [19]. Research highlights that health professional leaders have the power to influence how young professionals are conducting SDL, by e.g., supporting their protected time, or actively recommending which tools to use, which may alleviate some of these challenges.

The third and fourth research questions focus on what strategies and/ or digital tools experts use to navigate their learning environment. Many professionals avoid new technologies as they lack technological knowledge or time to become familiar in their use [34]. Many of the most experienced surgeons believed it was too late to adopt the latest tools, as they had not kept up with evolving technology. Recent research [35] suggests many professionals tend to stick to known, habitually safe tools as they believe themselves incapable of navigating the complexities of a new digital tool [35]. This is in line with the research on the importance of raising digital awareness from an early point [36] and to promote which digital tools are available to support SDL [33].

### Limitations

There are a number of limitations which should be considered in interpreting the findings of this present study. Perhaps most importantly, in the process of data analysis there could be issues with bias and the robustness of data interpretation. To reduce the risk of such biases and increase reliability, we correlated the stability of responses through a rigorous questionnaire development process and the main themes were coordinated with multiple coders. Reliability was enhanced by detailed field notes and a transcription of all interviews. The study focussed on the experience of neurosurgeons [37] however, the literature supports our findings in relation to other healthcare professionals particularly with similar demographics to neurosurgeons; we are confident our findings are robust and relevant. Lastly, due to the rapid increase of information and advancement in the use of digital technology, it should be considered that the findings of this study provide a snapshot in time and as the use of advanced technology becomes more mainstream, this study could present as an important reference point for comparison for future work.

## Conclusion

This study sought to investigate how neurosurgeons maintain and update their professional knowledge in an SDL context. The differences between less and more experienced neurosurgeons in the strategies they employ to conduct SDL and to select relevant high-quality sources of information is a key finding of this study. Both more and less experienced surgeons agree that they would benefit from a shared specialist platform for registered health professionals, to highlight landmark papers and allow more experienced surgeons to share their wisdom and maintain their knowledge base. Furthermore, less experienced surgeons are severely impacted by the unrealistic number of articles to read, conducting most of their SDL outside of working hours. There is also a divide in relation to the use of digital tools with less experienced surgeons more likely to make use of such technology versus their senior peers. To prevent a widening digital divide, awareness of what digital tools are available for professionals and how they support SDL needs to be raised.

The question now is how to follow-up on this study. The recent increase and level of SDL training and skill development in medical education can be anticipated to have had an impact on how recently qualified health professionals adopt new strategies and adapt to new technologies to facilitate their SDL. Future research should explore the potential impact of such training particularly in relation to SDL and skill development in the context of the powerful artificial intelligence (AI) tools such as ChatGPT or Google Gemini.

## Data Availability

The datasets used and/or analyzed during the current study are available from the corresponding author on reasonable request.
